# Agaro-Oligosaccharide Supplementation Alters the Gut Microbiota, Revealing Potential Agaro-Oligosaccharide-Utilizing Taxa in Healthy Japanese Adults

**DOI:** 10.3390/biomedicines14051112

**Published:** 2026-05-14

**Authors:** Natasia Hoshiba, Tadashi Fujii, Rina Yagasaki, Toshiyuki Ochi, Katsuhiro Shiba, Hideaki Takahashi, Kohei Funasaka, Eizaburo Ono, Yoshiki Hirooka, Takumi Tochio, Koji Karasawa

**Affiliations:** 1 Research & Development Department, Ina Food Industry, Co., Ltd., Ina 399-4498, Nagano, Japan; na-hoshiba@kantenpp.co.jp (N.H.); ri-yagasaki@kantenpp.co.jp (R.Y.); t-ochi@kantenpp.co.jp (T.O.); kshiba@kantenpp.co.jp (K.S.); 2 Department of Gastroenterology and Hepatology, Fujita Health University, Toyoake 470-1192, Aichi, Japan; tadashi.fujii@fujita-hu.ac.jp (T.F.); hideaki.takahashi.xk@fujita-hu.ac.jp (H.T.); k-funa@fujita-hu.ac.jp (K.F.); eizaburo.ono@fujita-hu.ac.jp (E.O.); yoshiki.hirooka@fujita-hu.ac.jp (Y.H.); t-tochio@fujita-hu.ac.jp (T.T.); 3 Department of Medical Research on Prebiotics and Probiotics, Fujita Health University, Toyoake 470-1192, Aichi, Japan; 4 BIOSIS Lab. Co., Ltd., Toyoake 470-1192, Aichi, Japan

**Keywords:** agaro-oligosaccharides, prebiotic, gut microbiota, *Ruminococcus gnavus*, *Bacteroides uniformis*, 3,6-anhydro-L-galactose cycloisomerase, healthy Japanese adults, open-label pilot study

## Abstract

**Background:** Agaro-oligosaccharides (AOS) have been shown to modulate the gut microbiota in in vitro and animal studies; however, human evidence remains scarce. **Methods:** Herein, we conducted a four-week open-label, single-arm, non-randomized pilot trial in 18 healthy Japanese adults to examine the association of AOS intake at 200 mg/day with gut microbiota composition and bowel condition. Fecal samples collected before and after the intervention were analyzed using QIIME2-based 16S rRNA sequencing, and bowel condition was assessed with the Bristol Stool Form Scale. This study was registered in the UMIN Clinical Trials Registry (UMIN000056992). **Results:** AOS intake was not associated with significant changes in bowel condition. Gut microbiota analysis showed no significant alterations in overall community structure but revealed taxon-specific trends in the relative abundance of several bacterial taxa. Notably, nominal changes were observed in the abundance of the *Ruminococcus gnavus* group and *Bacteroides uniformis* after the intervention. In addition, quantitative PCR analysis showed an increase in 3,6-anhydro-L-galactose cycloisomerase (ACI) gene abundance after the intervention. **Conclusions:** These findings suggest that, in this exploratory pilot study, AOS intake was associated with a taxon-specific pattern in the gut microbiota. Further randomized controlled studies are needed to clarify the microbiota-related effects of AOS in humans.

## 1. Introduction

Agar and its oligosaccharide derivatives have attracted considerable interest owing to their functional properties and potential health benefits, with applications spanning foods, nutraceuticals, and biomedical materials [1,2,3]. Among these compounds, agaro-oligosaccharides (AOS) are short-chain carbohydrates produced by the partial hydrolysis of agar, a polysaccharide extracted from red macroalgae composed of alternating D-galactose and 3,6-anhydro-L-galactose (AHG) residues linked by β-1,4 and α-1,3 glycosidic bonds. Owing to these unique linkages, AOS are considered resistant to digestion by host enzymes [4] and are therefore likely to reach the colon largely intact. As a result, AOS are increasingly recognized as functional dietary components whose biological effects are mediated through interactions with the gut microbiota [1].

In recent years, AOS have gained attention as bioactive compounds with reported prebiotic, immunomodulatory, anti-inflammatory, antioxidant, and antitumor activities. Prebiotics are defined as non-digestible dietary substrates that are selectively utilized by host microorganisms, thereby conferring health benefits [5,6]. However, most conventional prebiotics, such as fructans and galacto-oligosaccharides, broadly stimulate beneficial bacterial taxa, mainly *Bifidobacterium* and lactobacilli [7]. Although generally favorable, these substrates often lack taxonomic and functional specificity and have limited capacity to modulate microbial populations or metabolic activities associated with intestinal inflammation or ecological niche differentiation.

These limitations have prompted growing interest in the development of more “selective” prebiotics that shape gut microbial ecology by enriching distinct metabolic traits rather than globally enhancing bacterial growth. Several selective properties of AOS have been reported in vitro, including the growth inhibition of *Ruminococcus gnavus*, a mucin-degrading species frequently associated with inflammatory bowel disease (IBD) [8], as well as *Fusobacterium nucleatum*, while sparing beneficial microbial groups, such as *Bifidobacterium* and members of the order Lactobacillales [9]. These findings suggest that AOS may exert targeted effects on gut microbial composition and function. However, whether such selective effects observed under in vitro conditions translate into measurable shifts in microbial occupancy and functional potential in the human gut remains largely unknown.

Members of the genus *Bacteroides* dominate the human gut microbiota and are distinguished by their capacity to degrade diverse complex polysaccharides through specialized polysaccharide utilization loci (PULs) [10]. Certain *Bacteroides* species, including *Bacteroides plebeius*, harbor agar-specific PULs that encode extracellular agarases and intracellular enzymes required for the metabolism of agar-derived sugars [11,12]. These gene clusters enable the conversion of agarose into AOS, such as agarobiose, and subsequently into monosaccharides, including AHG, a rare sugar that is poorly metabolized by most gut microbes.

The complete assimilation of AHG requires a dedicated catabolic pathway in which 3,6-anhydro-L-galactose cycloisomerase (ACI) plays a central role by converting 3,6-anhydro-L-galactonate into downstream intermediates that can enter the central metabolism [13,14]. ACI has been structurally and biochemically characterized in agarolytic marine bacteria, where it is essential for growth on agar-derived substrates [15]. Genomic analyses have identified ACI homologs in several gut *Bacteroides* species, suggesting that these bacteria possess the metabolic capacity to fully utilize AOS once agar-derived oligosaccharides reach the colon [12,16]. Nonetheless, whether dietary exposure to AOS in humans can modulate the abundance of agar-degradation-related genes, including the ACI gene, or alter the relative occupancy of agar-utilizing *Bacteroides* within the gut microbiome, remains unclear.

Given that prebiotic efficacy is increasingly understood in terms of functional gene enrichment rather than taxonomic shifts alone, determining how AOS consumption influences both microbial community composition and metabolic potential in vivo is essential. In particular, whether AOS intake can simultaneously suppress potentially detrimental taxa identified in vitro and enhance agar-degrading capacity within *Bacteroides* populations has not yet been investigated in human intervention studies. Addressing this gap will provide new insight into how marine-derived oligosaccharides shape gut microbial functions and clarify the role of AOS as selective, function-targeted prebiotics in the human intestine.

## 2. Materials and Methods

### 2.1. Study Design and Participants

This study was an open-label, single-arm, non-randomized pilot intervention study designed to evaluate the association of AOS administration with bowel function and the gut microbiota in healthy Japanese adults. Reporting of the study followed the Transparent Reporting of Evaluations with Nonrandomized Designs (TREND) statement [17]. Eighteen participants (12 males and 6 females), aged 25 to 49 years, were recruited. Participants were recruited from employees of Ina Food Industry Co., Ltd. through an internal call for volunteers. Recruitment was conducted at Ina Food Industry Co., Ltd. in Japan. Eligibility criteria included no history of chronic gastrointestinal disorders, no antibiotic use within the past three months, and a self-reported normal bowel movement frequency.

Participants were excluded if they had any significant medical condition, prior abdominal surgery, were pregnant or breastfeeding, or had ongoing therapy with medications known to affect the gut microbiota (including the use of antibiotics, anti-diarrheal, or antihypertensive medications). Participants were also excluded if they had regularly consumed probiotics or prebiotics in the 2 months prior to enrollment or followed selective/restricted diets or used dietary replacements. None of the participants were taking regular prescription medications. All participants were instructed to refrain from consuming other probiotic or prebiotic products during the intervention period. No additional standardized dietary advice was provided, and dietary intake was not monitored or recorded during the intervention period.

All participants provided written informed consent prior to enrollment. The study was conducted in accordance with the Declaration of Helsinki and was approved by the Ethics Committee of Okutoeru LLC (Committee Number: 24000113) on 29 July 2024. The study was registered in the UMIN Clinical Trials Registry (UMIN000056992). In the registry, the anticipated trial start date was 30 July 2024, and the public release date was 10 February 2025. The delayed public release was related to considerations regarding patent application and intellectual property protection. Because this was an exploratory pilot study, no formal sample size calculation was performed. The analysis population was the full analysis set (FAS), defined a priori as all participants who received the AOS intervention and provided both baseline and post-intervention fecal samples.

### 2.2. Intervention

Participants consumed a specially prepared, non-commercial powdered test food product containing 200 mg of AOS, with maltodextrin as an excipient, per day for four consecutive weeks. The test food was manufactured by Ina Food Industry Co., Ltd. for research use. The powder was dissolved in water or other beverages and taken orally once daily. The test food and intake instructions were provided to the participants by the study staff. Participants consumed the test food at home once daily during the intervention period. Compliance was monitored through weekly follow-ups and self-reported intake logs. The daily dose of 200 mg was selected for this exploratory pilot study with reference to previous human data at the same intake level, which indicated that daily intake of AOS was well tolerated in healthy adults [18].

### 2.3. Assessment of Bowel Habits

Stool form was assessed using the 7-point Bristol Stool Form Scale (BSFS), a validated self-administered questionnaire widely applied in dietary intervention studies (e.g., Yamamoto et al., 2021 [19]). Bowel habits were recorded throughout the intervention period using standardized weekly logs. Participants reported stool frequency, consistency (based on the BSFS), and ease of defecation. For the main analysis, baseline data were compared with values obtained after the 4-week intervention to evaluate changes in bowel condition.

### 2.4. Fecal Sample Collection and Microbiota Analysis

Fecal samples were collected at baseline and after four weeks of AOS intake. Genomic DNA was extracted using a QIAamp PowerFecal Pro DNA Kit (QIAGEN, Hilden, Germany) according to the manufacturer’s instructions, following a previously described protocol [20]. Recovered genomic DNA was used as a template for the amplification of the V3-V4 hypervariable regions of the microbial 16S ribosomal RNA (rRNA) gene using the primers 341F (5′-CCTACGGGNGGCWGCAG-3′) and 805R (5′-GACTACHVGGGTATCTAATCC-3′) [21]. Paired-end sequencing was performed using the Illumina MiSeq platform (Illumina, San Diego, CA, USA) at Bioengineering Lab. Co., Ltd. (Sagamihara, Japan), as described previously [22].

Microbiota profiling was performed with QIIME 2 version 2024.5. Demultiplexed paired-end sequence data were denoised with DADA2 [23] to generate amplicon sequence variants (ASVs). The ASVs were aligned with MAFFT [24], and a phylogenetic tree was constructed using FastTree2 [25].

Alpha-diversity metrics (Shannon index, Simpson’s index, species richness, and species evenness), beta-diversity metrics (Bray–Curtis dissimilarity), and principal coordinate analysis (PCoA) were calculated using q2-diversity after rarefaction. The Silva reference database (release 138; 99% reference sequences) [26] was trained and used for taxonomy assignment using the q2-feature-classifier classify-sklearn naïve Bayes method [27].

Functional prediction of microbial enzymes and metabolic pathways was performed as an exploratory analysis using PICRUSt2 [28] based on the ASV abundances and phylogenetic placement.

### 2.5. Primer Design

To develop a primer set capable of collectively detecting the gene encoding 3,6-anhydro-L-galactonate cycloisomerase (ACI) within the agarose-metabolizing gene cluster of *Bacteroides* species, homologous sequences were identified using BLASTp on the NCBI BLAST web server. The ACI protein from *Bacteroides uniformis* NBRC 113350 (GAA5538643.1), obtained from NITE Biological Resource Center (NBRC, Kisarazu, Japan), and encoded on plasmid pBUN1, was used as the query sequence, and the search was performed against the NCBI database (https://www.ncbi.nlm.nih.gov/, accessed on 6 December 2025).

Among homologs showing >80% pairwise amino acid identity, a region conserved in *Bacteroides* species but divergent from Rikenellaceae bacteria was selected for primer design.

The forward (5′-AAATGTAAGGCATATTGTGGAGGAATAGACCT-3′) and reverse primers (5′-CAGGATTTTCGCGACCAATCTTTATTTTAA-3′) were designed to anneal to the positions around 400–431 bp and 498–526 bp, respectively, of the ACI homolog (Supplementary Figure S1).

### 2.6. Quantitative PCR Analysis

The abundances of the ACI and total bacterial 16S rRNA genes were quantified via qPCR using a QuantStudio^TM^ 3 Real-Time PCR System (Thermo Fisher Scientific, Waltham, MA, USA). Reaction mixtures were prepared using a PowerTrack^TM^ SYBR Green Master Mix (Thermo Fisher Scientific) according to the manufacturer’s instructions. Gene-specific primers for ACI (described above) and universal primers for the bacterial 16S rRNA gene (F_Bact 1369 and R_Prok 1492 [29]) were used.

The qPCR amplification protocol consisted of an initial denaturation step at 95 °C for 2 min, followed by 40 cycles of denaturation at 95 °C for 10 s, annealing at 60 °C for 15 s, and extension at 72 °C for 15 s. A final extension step at 72 °C for 1 min was included in all reactions.

The abundance of the ACI gene was normalized to the total bacterial 16S rRNA gene copy number to account for differences in total bacterial load. Absolute quantification of the ACI gene was performed using a standard curve generated from serial dilutions of PCR-amplified ACI gene fragments derived from *B. uniformis* NBRC 113350.

### 2.7. Next-Generation Sequencing (NGS) Analysis of Amplified ACI Fractions

As an exploratory analysis, NGS was performed on amplified ACI gene fragments obtained from the three samples exhibiting the highest ACI gene abundance as determined by qPCR. The amplicons were purified using a Wizard SV Gel and PCR Clean-Up System (Promega, Madison, WI, USA) and eluted in 30 µL of the supplied elution buffer. The purified DNA was subsequently used as a template for a secondary PCR to incorporate Illumina adapter sequences.

The primer set used for this amplification consisted of the forward primer ACI_forNGS_F

(5′-ACACTCTTTCCCTACACGACGCTCTTCCGATCTaaatgtaaggcatattgtggaggaatagacct-3′)

and the reverse primer ACI_forNGS_R

(5′-GTGACTGGAGTTCAGACGTGTGCTCTTCCGATCTcaggattttcgcgaccaatctttattttaa-3′).

In these primers, the sequences in lowercase correspond to the ACI gene-specific primers used for qPCR, whereas the sequences in uppercase represent the Illumina adapter regions required for sequencing.

The secondary PCR reaction was performed using Quick Taq HS DyeMix (Toyobo, Osaka, Japan) with a final primer concentration of 3.2 pM. The thermal cycling conditions consisted of an initial denaturation step at 94 °C for 2 min, followed by a cycle at 68 °C for 15 s and 72 °C for 15 s, then nine cycles of denaturation at 95 °C for 5 s, annealing at 68 °C for 15 s, and extension at 72 °C for 15 s. A final extension step was performed at 72 °C for 1 min.

Following amplification, the PCR products were purified again using the Wizard SV Gel and PCR Clean-Up System (Promega, Tokyo, Japan) and eluted in 50 µL of the supplied elution buffer. The purified amplicons were subjected to paired-end sequencing (2 × 300 bp) on a MiSeq platform (Illumina) by Bioengineering Lab Co., Ltd.

### 2.8. Fecal Short-Chain Fatty Acid Analysis

Short-chain fatty acids (SCFAs) in fecal samples were analyzed as described previously [30], with minor modifications. Briefly, fecal samples (20–50 mg) were homogenized in phosphate-buffered saline, and SCFAs (acetate, propionate, and butyrate) were derivatized with 2-nitrophenylhydrazide using a Short- and Long-Chain Fatty Acid Analysis Kit (YMC, Kyoto, Japan). Derivatized SCFAs were extracted with diethyl ether and quantified via high-performance liquid chromatography using a Nexera XL system (Shimadzu Corporation, Kyoto, Japan) equipped with a YMC-Pack FA column, with detection at 400 nm. SCFA concentrations were expressed as µmol/g wet feces and were not normalized for stool-water content.

### 2.9. Statistical Analyses

Statistical analyses were conducted using GraphPad Prism version 10 (GraphPad Software, San Diego, CA, USA) and R version 4.5.1 (R Core Team, 2020). For within-participant comparisons (baseline vs. week 4), Wilcoxon signed-rank tests were used. Differences in beta diversity were assessed using permutation multivariate analysis of variance (PERMANOVA) based on the Bray–Curtis dissimilarity.

Results are presented as individual values with summary statistics (mean ± SD or median [IQR], as appropriate). A two-sided *p*-value < 0.05 was considered statistically significant. For taxon-level comparisons involving multiple tests, *p*-values were adjusted using the Benjamini–Hochberg false discovery rate (FDR) procedure. The detailed statistical methods and specific tests applied for each analysis are described in the corresponding figure legends.

## 3. Results

### 3.1. Participants and Baseline Characteristics

**Figure 1:**
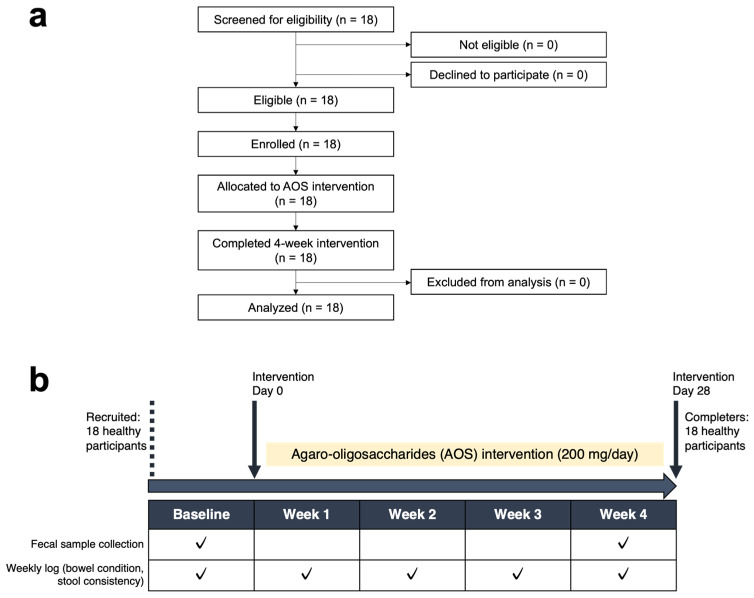
Participant flow and study design of the 4-week AOS intervention: (**a**) Flow diagram of participant screening, eligibility assessment, enrollment, allocation, follow-up, and analysis. (**b**) Study design of the 4-week AOS intervention. Fecal samples were collected at baseline and week 4 to assess gut microbiota and short-chain fatty acids (SCFAs). Participants recorded their weekly bowel condition and stool consistency throughout the intervention period.

The participant flow diagram is shown in Figure 1a, and the study design of the AOS intervention is shown in Figure 1b. Participant recruitment began in late July 2024. A total of 18 participants were enrolled and began the 4-week intervention on 30 July 2024. All 18 participants received the intervention, completed the 4-week intervention, and were included in the FAS. All participants in this analysis set consumed 200 mg/day of AOS for 4 weeks as instructed.

The baseline characteristics of the participants are summarized in Table 1. The cohort included 12 males (66.7%) and 6 females (33.3%), with a mean age of 38.3 ± 6.6 years. The mean height, weight, and BMI of the participants were 169.2 ± 8.1 cm, 61.2 ± 9.6 kg, and 21.3 ± 2.5 kg/m^2^, respectively. Regarding lifestyle factors, 72.2% were non-smokers, 50.0% were current alcohol consumers, and 27.7% reported regular exercise.

### 3.2. Bowel Condition and Stool Characteristics

**Figure 2:**
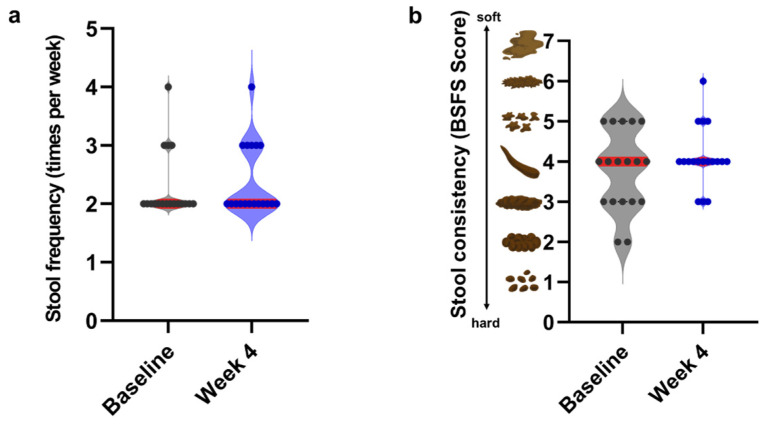
Effects of AOS intake on stool characteristics in healthy adults. Stool frequency (**a**) and consistency (**b**) before and after the 4-week AOS intervention are shown as violin plots with the individual data points overlaid (*n* = 18). Each dot represents an individual participant, and the horizontal red lines indicate the median values. No significant differences were observed between time points as determined by the Wilcoxon signed-rank test.

Stool frequency and consistency were recorded throughout the 4-week AOS intervention period using a self-administered questionnaire, and the main analysis compared baseline and week 4 values. After 4 weeks of AOS intake, no significant changes were observed in stool frequency (median [IQR], baseline: 2.0 [2.0–2.25] vs. week 4: 2.0 [2.0–3.0], *n* = 18, *p* = 0.2667) or BSFS score (median [IQR], baseline: 4.0 [3.0–5.0] vs. week 4: 4.0 [4.0–4.25], *n* = 18, *p* = 0.5000), as determined using the Wilcoxon signed-rank test. Violin plot analysis further demonstrated that, at the cohort level, the distribution of BSFS scores after the intervention was more tightly clustered around intermediate values compared with that at baseline, despite no change in the median score (Figure 2). This pattern was consistent with a reduced dispersion of stool consistency across participants rather than a shift in central tendency.

Consistent with these findings, most participants maintained their baseline bowel habits throughout the study. No statistically significant differences were detected in fecal SCFA concentrations (acetate, propionate, and butyrate; Supplementary Figure S2), and no gastrointestinal adverse events were reported.

### 3.3. Effects of AOS on the Gut Microbiota

#### 3.3.1. Alpha and Beta Diversity of Gut Microbiota

To evaluate whether AOS intake was associated with changes in gut microbiome diversity, we profiled the fecal microbial communities of 18 participants using 16S rRNA gene sequencing before and after the intervention.

**Figure 3:**
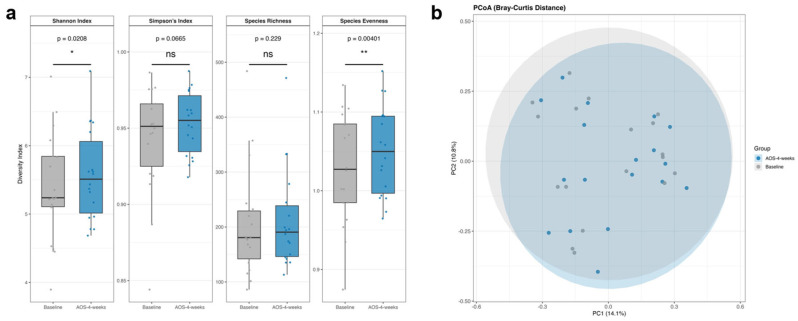
Alpha and beta diversity of the gut microbiota before and after 4 weeks of AOS intervention: (**a**) Alpha-diversity metrics (Shannon index, Simpson’s index, species richness, and evenness) show significant changes in Shannon index and species evenness (Wilcoxon signed-rank test). (**b**) Beta diversity (PCoA based on Bray–Curtis dissimilarity) shows no significant differences between pre- and post-intervention samples (PERMANOVA). Asterisks indicate statistical significance (* *p* < 0.05, ** *p* < 0.01); ns, not significant.

Alpha diversity was assessed using the Shannon index, Simpson’s index, species richness, and species evenness (Figure 3a). The Shannon index (*p* < 0.05) and species evenness (*p* < 0.01) differed significantly between baseline and 4 weeks of AOS intervention, whereas the other alpha-diversity metrics did not show significant differences.

Beta diversity was evaluated using Bray–Curtis dissimilarity and visualized using PCoA (Figure 3b). The microbial community structures before and after AOS intake largely overlapped, with no significant separation detected (permutational multivariate analysis of variance [PERMANOVA], R^2^ = 0.0068, *p* = 1.000).

Taken together, the 4-week AOS intervention was associated with minor changes in selected alpha-diversity metrics, whereas the overall community structure remained stable.

#### 3.3.2. Distribution and Diversity of the Gut Microbiota at the Phylum, Genus, and Species Levels

**Figure 4:**
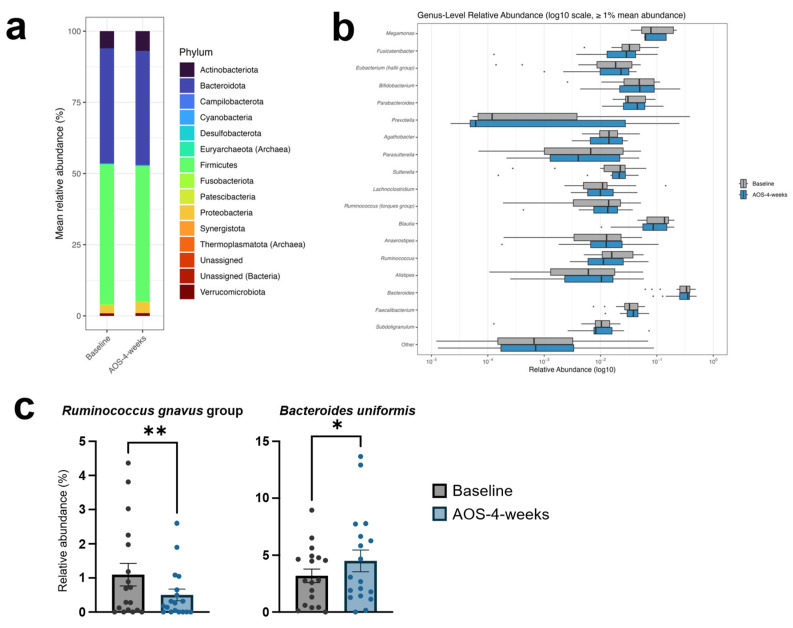
Taxonomic profiles of the gut microbiota following the 4-week AOS intervention: (**a**) Relative abundance of dominant bacterial phyla. (**b**) Dominant genera (mean relative abundance ≥ 1%) presented on a log_10_ scale, with bullets indicating outliers. (**c**) Species-level taxa showing nominal changes after AOS intake, including the *R. gnavus* group and *B. uniformis*. Taxon-level comparisons were assessed using the Wilcoxon signed-rank test. Asterisks indicate nominal significance based on unadjusted *p*-values (* *p* < 0.05, ** *p* < 0.01). None of the observed genus- or species-level changes remained statistically significant after FDR correction.

The gut microbiota of the participants was dominated by the phyla Firmicutes and Bacteroidetes, followed by Actinobacteria and Proteobacteria (Figure 4a). At the genus level, no dominant genera (relative abundance ≥ 1%) showed a significant shift after AOS intake (Figure 4b). However, four lower-abundance genera—*Ruminococcus*, *Marvinbryantia*, *Intestinibacter*, and Lachnospiraceae DTU089—showed nominal differences following the intervention (Wilcoxon signed-rank test, Supplementary Table S1), although none remained statistically significant after FDR correction. At the species level, most taxa showing nominal shifts were unresolved within QIIME2 and annotated as *Genus* sp. (Supplementary Table S2). Notably, the abundance of the *R. gnavus* group showed a nominal decrease, whereas that of *B. uniformis* showed a nominal increase after AOS intake (Figure 4c); however, these changes did not remain statistically significant after FDR correction.

Overall, these findings suggest that AOS intervention was associated with taxon-specific trends, while the broader community composition remained largely stable.

### 3.4. Exploratory Functional Predictions of Galactose Metabolism Following AOS Intake

Functional predictions generated using PICRUSt2 were applied as an exploratory analysis to assess potential metabolic trends in the gut microbiota following AOS intake. This analysis suggested trends toward increased relative abundances of predicted enzyme activities associated with galactose metabolism, including EC 2.7.1.58 (2-keto-3-deoxy-galactonokinase), which catalyzes the phosphorylation of 2-keto-3-deoxy-galactonate (KDGal) into 2-keto-3-deoxy-6-phosphogalactonate (KDPGal), and EC 4.1.2.21 (2-keto-3-deoxy-6-phosphogalactonate), a lyase that cleaves KDPGal into pyruvate and glyceraldehyde-3-phosphate (GAP) (Table 2). Although the predicted abundance of KDPGal aldolase increased approximately 16-fold relative to baseline, this change did not remain statistically significant after Benjamini–Hochberg correction.

### 3.5. qPCR-Based Quantification of ACI Gene Levels

AOS are composed of D-galactose and 3,6-anhydro-L-galactose (AHG). In this study, we quantified ACI gene abundance using a targeted qPCR assay designed to detect ACI homolog genes. ACI is involved in the initial step of AHG metabolism. Based on sequence similarity searches of publicly available genomes in the NCBI database, the *ACI* gene appears to be present in only a limited number of *B. uniformis* strains and appears to be rare across the genus *Bacteroides* (Supplementary Figure S1). Primers were designed to collectively detect ACI gene-positive *B. uniformis* strains as well as other *Bacteroides* species harboring the *ACI* gene, and qPCR was used to compare ACI gene abundance before and after AOS ingestion.

**Figure 5:**
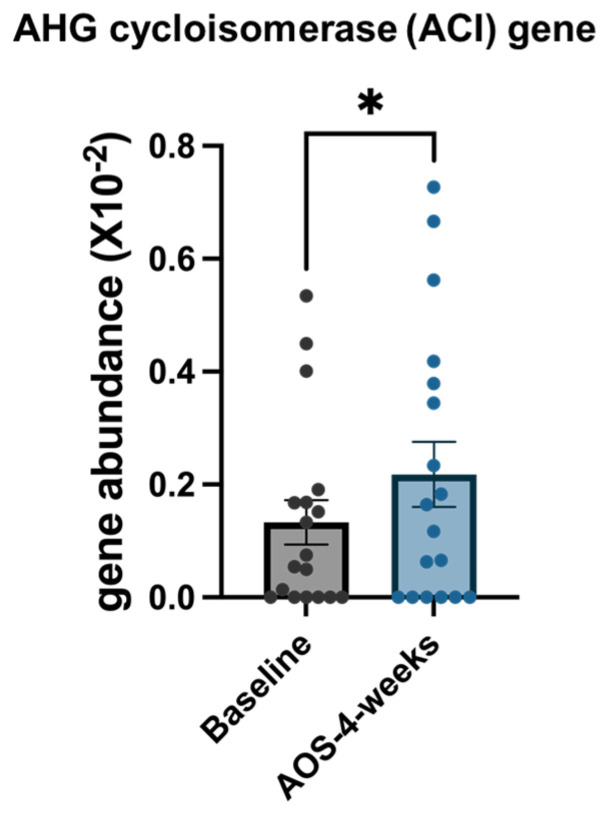
qPCR-based quantification of ACI gene abundance before and after AOS intervention. Significance levels: *p* < 0.05 (*).

qPCR analysis revealed a significant increase in ACI gene abundance after the 4-week intervention compared with that at baseline (*p* = 0.0122) (Figure 5).

### 3.6. NGS Analysis of Amplified ACI Gene Fragments

As an exploratory analysis to further evaluate the specificity of the primer set targeting the ACI gene, NGS was performed on PCR-amplified ACI gene fragments obtained from the three samples exhibiting the highest ACI gene abundance in the qPCR analysis. A total of 120,309 reads were generated, and the characteristics of the resulting ASVs are summarized in Supplementary Table S3.

BLAST analysis indicated that all ASVs showed high sequence similarity to the ACI gene of *B. uniformis* NBRC 113350 (accession No. AP019725.1), with 0–2 mismatches over a 65-bp alignment. Within the limits of the reference database and the short alignment length, no other *Bacteroides* species exhibited comparable sequence similarity in the BLAST results.

## 4. Discussion

In this study, four-week oral AOS administration in humans was associated with minor changes in within-sample diversity (e.g., Shannon index and evenness), while overall community structure (β-diversity), bowel habits, or stool characteristics remained stable in healthy adults. Despite this overall stability, AOS intake was associated with taxon-specific trends, including nominal changes in the *R. gnavus* group and *B. uniformis*. These findings suggest a selective, taxa-specific pattern in the gut microbiota rather than broad compositional shifts.

The apparent decrease in the abundance of the *R. gnavus* group is of interest, as this taxon has been frequently associated with mucosal inflammation, including inflammatory bowel disease. The elevated abundance of the *R. gnavus* group has been linked to disease activity and inflammatory responses [8], and the apparent decrease observed in the present study may be consistent with a less pro-inflammatory microbial profile, although no clinical inflammatory outcomes were assessed. These findings are broadly consistent with previous in vitro work by Fujii et al. [9], which demonstrated that AOS inhibited the growth of *R. gnavus* and *F. nucleatum* while sparing beneficial taxa. Notably, in the present human study, AOS intake was not associated with clear changes in the abundance of beneficial bacteria, including Bifidobacteriaceae and Lactobacillaceae (Supplementary Figure S3). Together, these observations suggest that AOS intake may be associated with selective modulation of inflammation-associated members of the gut microbiota without clear disruption of beneficial populations.

Unlike many prebiotic or probiotic interventions that produce minimal or inconsistent effects in healthy individuals [31], the present study suggested taxon-specific microbiota trends following AOS intake in healthy adults. This specificity may be related to the structural characteristics of AOS, which are derived from agarose and are not readily utilized by most gut commensals, thereby restricting metabolic accessibility to a limited subset of bacteria [32]. Moreover, because AOS are relatively resistant to host digestive enzymes, they are likely to transit the upper gastrointestinal tract largely intact and remain available for microbial utilization in the colon [33,34]. In contrast, many established prebiotics are rapidly fermented and typically require gram-scale daily doses to elicit measurable effects [35,36,37]. The present findings therefore raise the possibility that AOS intake may be associated with taxon-specific microbiota trends even at comparatively low daily doses (mg range), although further studies are required to clarify dose–response relationships and colonic availability.

We also observed a nominal increase in the relative abundance of *B. uniformis*, a species known for its capacity to degrade complex polysaccharides. Notably, total fecal SCFA concentrations remained unchanged following AOS intake, suggesting that the observed taxonomic changes were not accompanied by detectable alterations in overall fermentative output. This may indicate that AOS-associated microbial responses are linked more to substrate- or pathway-specific metabolic adaptations than to global enhancement of carbohydrate or SCFA fermentation.

Because AOS are composed of D-galactose and AHG, a structural residue characteristic of agar-derived oligosaccharides, we next focused on microbial genes involved in AHG utilization. qPCR analyses revealed a significant increase in the abundance of a *Bacteroides*-specific *ACI* gene following AOS intake. ACI catalyzes the conversion of AHG-derived intermediates into KDGal. In parallel, PICRUSt2-based functional predictions suggested trends toward increased representation of enzymes involved in downstream KDGal catabolic pathways, including 2-keto-3-deoxy-galactonokinase and 2-keto-3-deoxy-6-phosphogalactonate aldolase. Although these predicted changes did not remain statistically significant after multiple-testing correction, KDGal is a well-established intermediate of the oxidative galactose (DeLey–Doudoroff) pathway [38,39] and has also been identified as a central intermediate in AHG catabolism [14]. Taken together, these findings may suggest a change in microbial genetic features potentially related to AOS metabolism, even in the absence of measurable changes in SCFA production.

Consistent with this interpretation, both NGS and qPCR analyses were consistent with the presence of *Bacteroides* taxa with putative AOS-metabolizing capacity. Together, these findings suggest that AOS intake may be associated with shifts toward bacterial populations potentially equipped with agar- and AHG-utilizing pathways, rather than broad stimulation of microbial growth.

Although these findings provide preliminary in vivo evidence consistent with prior in vitro observations, they do not demonstrate the direct utilization of AOS or AHG in the human gut. Functional confirmation will therefore require additional analyses, such as enzyme activity assays, metabolite tracing, or cultivation-based approaches. In this context, Pluvinage et al. (2018) demonstrated that the *B. uniformis* strain NP1 can degrade agarose through the activity of GH86 and GH16B, with the resulting oligosaccharides further processed into D-Galactose and AHG by GH117B and GH2C acting at the non-reducing end [12]. Targeted investigation of these enzymes in human intervention studies will be essential to elucidate the mechanisms underlying AOS degradation in the gut.

AHG is an anhydrous monosaccharide not found in terrestrial plants, while the genes involved in agar and AHG metabolism are predominantly found in marine bacteria. Prior studies have demonstrated that agarolytic and AHG-metabolizing pathways are widespread in marine microorganisms and that homologous genes can be detected in *Bacteroides* in the human gut, which were likely acquired through horizontal gene transfer associated with seaweed consumption [12,40,41,42]. The apparent presence of agar and AHG metabolism-related genes in a limited subset of *Bacteroides* strains in healthy Japanese individuals may be consistent with a similar evolutionary process, although this remains speculative and warrants further investigation.

Although no significant changes in median BSFS scores were observed following AOS intake, violin plot analysis indicated reduced interindividual variability, with values clustering around intermediate stool consistency. This pattern may be consistent with greater uniformity in stool consistency rather than a pronounced effect on bowel habits, although the underlying mechanism remains unclear. Collectively, these findings suggest that AOS intake may be associated with subtle changes in stool consistency distribution, while overall bowel habits remained stable. These observations may differ from patterns reported for broadly fermentable prebiotics, although further studies are needed to clarify their relevance to the functional effects of AOS.

From an implementation perspective, all 18 participants completed the 4-week intervention, and compliance was monitored through weekly follow-ups and self-reported intake logs. However, because intake was not directly observed and dietary intake was not recorded during the intervention period, the fidelity of implementation could not be assessed in greater detail.

Overall, these findings may be consistent with the emerging concept of AOS as a selective prebiotic. Rather than broadly stimulating classical beneficial taxa, AOS intake may be associated with changes in specific microbial populations, including apparent changes in inflammation-associated bacteria and bacteria with putative metabolic capacity for agar-derived substrates. Such function-targeted modulation may represent an alternative perspective for microbiota-directed dietary interventions, although its relevance to the maintenance of intestinal health remains to be clarified.

### Limitation of the Study

This study has several limitations. First, the single-arm, open-label design without a placebo control limits causal inference. Second, the study population was restricted to healthy Japanese adults aged 25–49 years, which may limit generalizability. In addition, because the study enrolled healthy participants with generally normal bowel habits, the potential for detecting changes in bowel-related outcomes may have been limited. Third, the sample size was modest, the intervention duration was relatively short for evaluating microbiota-related outcomes, and the study was not designed based on a formal sample size calculation. Finally, functional inferences were based on gene abundance and predictive analyses; direct assessment using shotgun metagenomics, metatranscriptomics, metabolomics, targeted investigation of agarolytic enzymes, or targeted in vitro experiments will be necessary to confirm the microbial pathways involved in AOS utilization. Future randomized, placebo-controlled studies with larger and more diverse cohorts, predefined primary endpoints, and appropriate sample size planning are warranted.

## Figures and Tables

**Table 1:** Baseline characteristics of participants enrolled in the AOS intervention study. Values are presented as the mean (standard deviation, SD) and percentage.

Variable	Study Population (*n* = 18)
Gender	
Female (%)	33.3
Male (%)	66.7
Mean age (years) (SD)	38.3 (6.6)
Mean height (cm) (SD)	169.2 (8.1)
Mean weight (kg) (SD)	61.2 (9.6)
Mean BMI (kg/m^2^) (SD)	21.3 (2.5)
Smoking	
Non (%)	72.2
Previously (%)	11.1
Current (%)	16.7
Alcohol	
Non (%)	44.4
Previously (%)	5.6
Current (%)	50.0
Exercise	
Non (%)	5.6
Previously (%)	66.7
Current (%)	27.7

**Table 2:** Predicted enzyme activities showing exploratory changes following AOS intervention based on PICRUSt2 analysis.

EC Number	Enzyme	FDR	log_2_ FC
EC:1.1.1.90	p-hydroxybenzyl alcohol dehydrogenase	0.201	3.66
EC:2.1.1.178	16S rRNA (cytosine1407-C5)-methyltransferase	0.201	1.05
EC:2.1.1.186	23S rRNA (cytidine2498-2′-O)-methyltransferase	0.201	1.18
EC:2.3.1.118	N-hydroxyarylamine O-acetyltransferase	0.201	1.02
EC:2.7.1.184	Sulfofructose kinase	0.201	1.92
EC:2.7.1.58	2-keto-3-deoxygalactonokinase	0.201	1.53
EC:3.1.2.28	1,4-dihydroxy-2-naphthoyl-CoA hydrolase	0.201	2.20
EC:4.1.2.21	2-keto-3-deoxy-6-phosphogalactonate aldolase	0.201	4.04
EC:4.2.1.80	2-keto-4-pentenoate hydratase	0.201	2.81
EC:4.2.1.83	4-oxalmesaconate hydratase	0.201	2.17

Enzyme activities were filtered using a nominal *p* < 0.01 and fold change > 2. *p*-values were adjusted using the Benjamini–Hochberg false discovery rate (FDR); none remained statistically significant after correction. Enzymes putatively related to galactonate catabolism are highlighted for reference.

## Data Availability

The raw 16S rRNA gene sequencing data generated in this study have been deposited in the NCBI Sequence Read Archive (SRA) under BioProject accession number PRJNA1390891 (http://www.ncbi.nlm.nih.gov/bioproject/1390891, accessed on 10 May 2026) and will be released upon publication.

## Supplementary Materials

The following supporting information can be downloaded at: https://www.mdpi.com/article/10.3390/biomedicines14051112/s1, Figure S1: Nucleotide sequence alignment of the ACI protein-encoding region from Bacteroides uniformis NBRC 113350 (GAA5538643.1, CDS Region in Nucleotide: BAABRW010000020.1 37271-38365 (+)) and its homologous genes; Figure S2: Fecal short-chain fatty acid (SCFA) concentrations before and after AOS intervention; Figure S3: Relative abundance of Bifidobacteriaceae and Lactobacillaceae in fecal samples before and after AOS intervention; Table S1: Genus-level nominal changes in gut microbiota composition following AOS intervention as assessed using the Wilcoxon signed-rank test; Table S2: Species-level nominal changes in gut microbiota composition following AOS intervention as assessed using the Wilcoxon signed-rank test; Table S3: Characteristics of ASVs derived from NGS analysis of PCR amplicons targeting the 3,6-anhydro-L-galactonate cycloisomerase gene.

## Abbreviations

The following abbreviations are used in this manuscript:

ACI	3,6-anhydro-L-galactonate cycloisomerase
AHG	3,6-anhydro-L-galactose
AOS	agaro-oligosaccharides
ASVs	amplicon sequence variants
BSFS	Bristol Stool Form Scale
FAS	full analysis set
FDR	false discovery rate
KDGal	2-keto-3-deoxy-galactonate
KDPGal	2-keto-3-deoxy-6-phosphogalactonate
NGS	next-generation sequencing
PCR	polymerase chain reaction
PCoA	principal coordinate analysis
PERMANOVA	permutational multivariate analysis of variance
qPCR	quantitative polymerase chain reaction
SCFAs	short-chain fatty acids
UMIN	University Hospital Medical Information Network
